# Optimizing expression of functional HIV envelopes in rVSV-ΔG vaccine vectors

**DOI:** 10.1186/1742-4690-9-S2-P342

**Published:** 2012-09-13

**Authors:** KJ Wright, M Yuan, A Wilson, C Boggiano, M Kemelman, P Tiberio, S Phogat, I Lorenz, S Hoffenberg, C Jurgens, C King, M Caulfield, C Parks

**Affiliations:** 1 IAVI, Brooklyn, NY, USA; 2 Sanofi Pasteur, USA; 3 Boehringer Ingelheim, USA

## Background

Our objective is to develop replicating recombinant vesicular stomatitis virus (rVSV) vectored HIV vaccine candidates that deliver membrane-bound trimeric HIV Env in a functional conformation.

## Methods

Using a combination of nucleotide sequence optimization and protein domain swapping, we have generated a panel of novel gene inserts for VSV vectors that encode chimeric HIV-1 and VSV glycoprotein immunogens (EnvG). A stable VERO cell line engineered to express human CD4 and CCR5 was used to rescue rVSV vectors in which the G gene was replaced with coding sequence for several different EnvG proteins.

## Results

Analysis of cells transfected with plasmid DNA expressing EnvG constructs revealed abundant cell surface expression of chimeric glycoproteins. The expressed proteins retained CD4-dependent membrane fusion activity, which is one of the main characteristics of functional HIV Env. The chimeric EnvG in which the signal peptide (SP), transmembrane (TM) and cytoplasmic tail (CT) domains of HIV Env were replaced with functionally related domains of VSV G were expressed efficiently and supported vector propagation to high titer specifically in CD4+CCR5+ cells. Flow cytometric analysis demonstrated that cell-surface expressed EnvG chimeras were recognized by a spectrum of HIV-1 specific broadly neutralizing monoclonal antibodies, including those that bind preferentially to the trimeric spike. Western blot analysis on purified viruses indicated that EnvG glycoproteins that contained the VSV G TM and CT were incorporated efficiently into VSV particles. Interestingly, an EnvG in which the Env MPER domain was replaced with membrane-proximal sequence from G was more effectively processed and incorporated into virus particles.

## Conclusion

Chimeric EnvG glycoproteins expressed efficiently from plasmid DNA and rVSV vectors in membrane-bound, fusion-competent conformation and displayed relevant HIV-1 broadly neutralizing antibody epitopes. Small animal studies are underway to assess Env-specific humoral immune responses elicited by rVSV vectors encoding EnvG immunogens.

